# Supplementary material for: Defect scattering can lead to enhanced phonon transport at nanoscale
Source: Nat Commun. 2024 Apr 17;15:3304. doi: 10.1038/s41467-024-47716-4 (PMC11024214; doi:10.1038/s41467-024-47716-4)
Supplement: Supplementary file 1 — Supplementary Information [file 41467_2024_47716_MOESM1_ESM.pdf]

Supplementary Information

Defect scattering can lead to enhanced phonon transport at nanoscale

Yue Hu[1,4], Jiaxuan Xu[1,2], Xiulin Ruan[3], and Hua Bao[1,2,*]

Note S1. Convergence test of MD Simulations

To check whether a cross-sectional area of 8×8-unit cells is sufficient, we further increase the cross-sectional area (up to 24×24-unit cells), as shown in Fig. S1. It can be seen that the results do not change, suggesting that phonon-boundary scattering does not play a significant role in the current system.

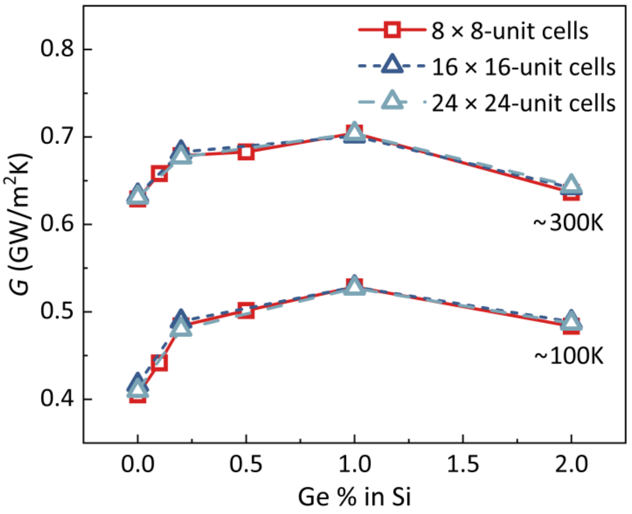

Fig. S1 | Thermal conductance from MD simulations for a cross-sectional area of 8×8, 16×16, and 24×24-unit cells.

We also eliminate fixed atoms in MD simulations and adopt periodic boundaries, as shown in Fig. S2. Without fixed atoms, there is also an increase in thermal conductance due to defect scattering (Fig. S3), indicating that fixed atoms have a negligible impact on the results.

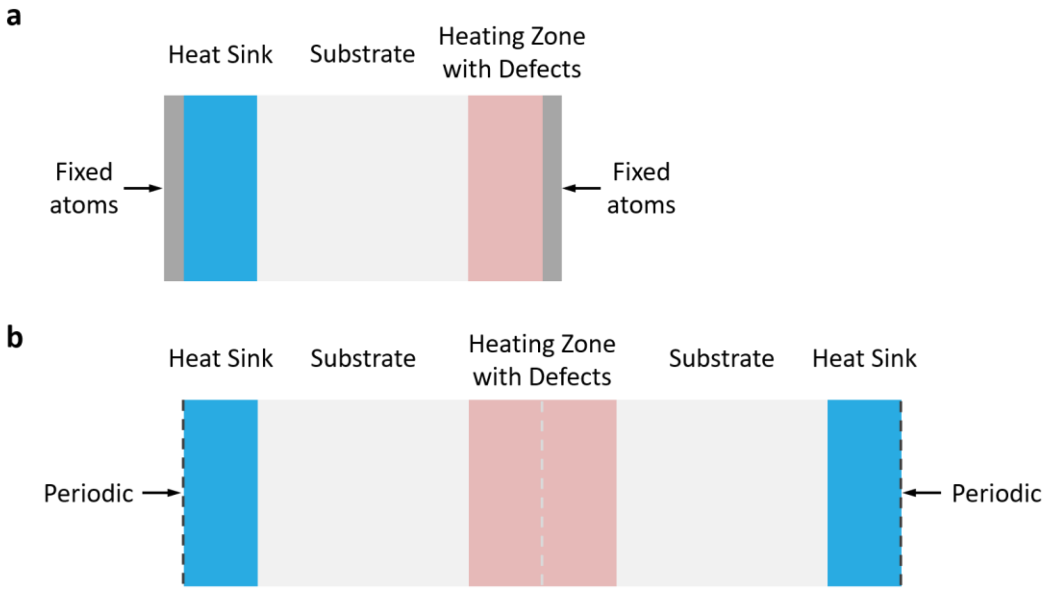

Fig. S2 | Simulation systems in MD. a Simulation system studied in MD in the manuscript. **b** Symmetrical simulation system with periodic boundaries instead of adopting fixed atoms in MD. The substrate is pure Si and the heating zone is Si with Ge impurities occupying random sites.

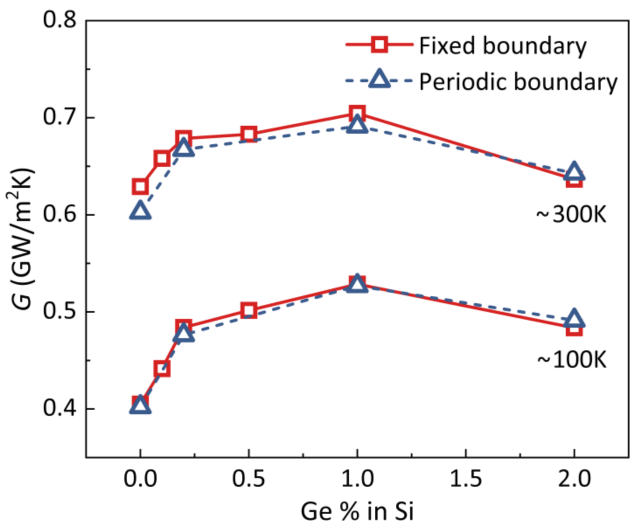

Fig. S3 | Thermal conductance results from MD for simulation systems shown in Fig. S2.

Note S2. Directional phonon energy flux at 300 K

At 300 K, due to the smaller phonon-phonon relaxation time [1], the phonon transport for pure Si is more diffusive than at 100 K, and the directional phonon nonequilibrium is smaller for pure Si (Fig. S4). Therefore, the thermal conductance enhancement is smaller at 300 K than at 100 K.

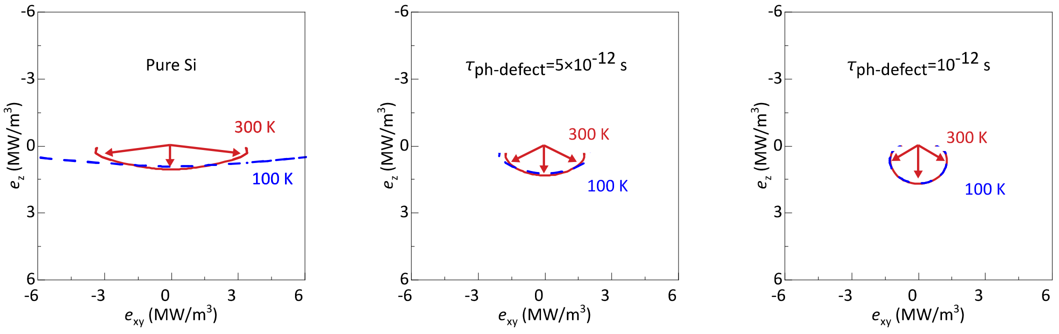

Fig. S4 | Directional phonon energy flux in the substrate at 300 K (simplified phonon BTE).

Note S3. Bulk thermal conductivity of Si

We carried out the first-principles calculations using the Quantum ESPRESSO package [2] to calculate the atomic forces and extract harmonic and third-order anharmonic interatomic force constants by fitting the relation between atomic forces and the displacements. A supercell of $4 \times 4 \times 4$ and the fourth nearest atom neighbor is considered to obtain the third-order anharmonic interatomic force constants. The thermal conductivity of Si is calculated based on the single-mode relaxation time approximation method. We use $60 \times 60 \times 60$ q-points for all temperatures to sample the Brillouin zone. The bulk thermal conductivity of Si for different temperatures for different temperatures obtained from first-principles calculations is shown in Fig. S5. Our results agree well with those in the literature [3].

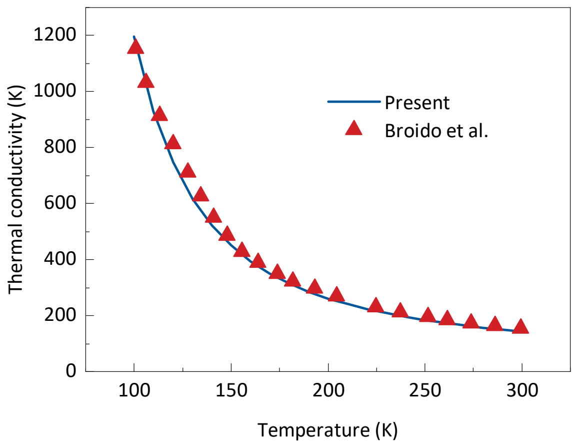

Fig. S5 | Bulk thermal conductivity of Si obtained from first-principles calculations. The symbols correspond to values in the reference [3].

Note S4. Phonon mean free path distribution of Si with Ge impurities

Figure S6 shows the phonon density of state (DOS) of Si with Ge impurities. It shows that the concentration has a minor impact on the DOS, most likely attributed to the low defect concentration. How different phonons contribute to the thermal transport can be analyzed from the phonon scattering rate or the phonon mean free path with different frequencies from first-principles calculations as shown in Fig. S7. For the pure Si, the phonon mean free path for all temperatures is generally larger than the length of the heating zone (10 nm) and the phonon transport is in the ballistic regime (compared with 10 nm in a-d). With decreasing temperature, the phonon mean free path increases and the phonon transport in the heating zone becomes more ballistic. One can see that by inducing defect scattering, the phonon mean free path decreases. When adding 2% Ge, the phonon mean free path is generally smaller than the length of the heating zone and the phonon transport is in the diffusive regime (compared with 10 nm in a-d).

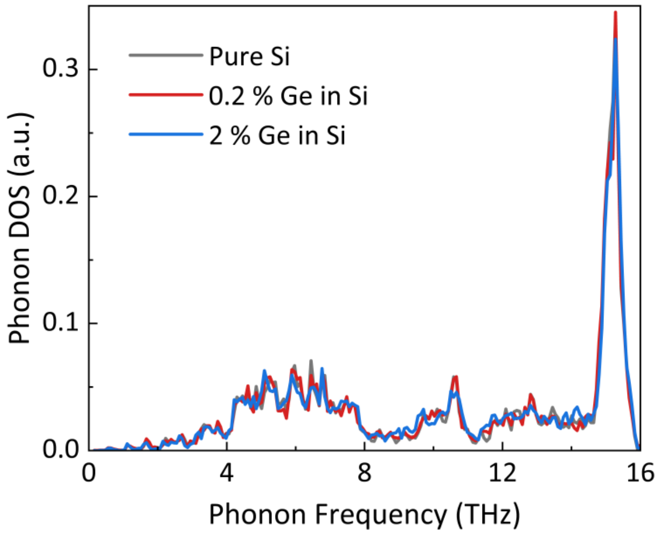

Fig. S6 | The phonon density of states for Si under different doped Ge concentrations.

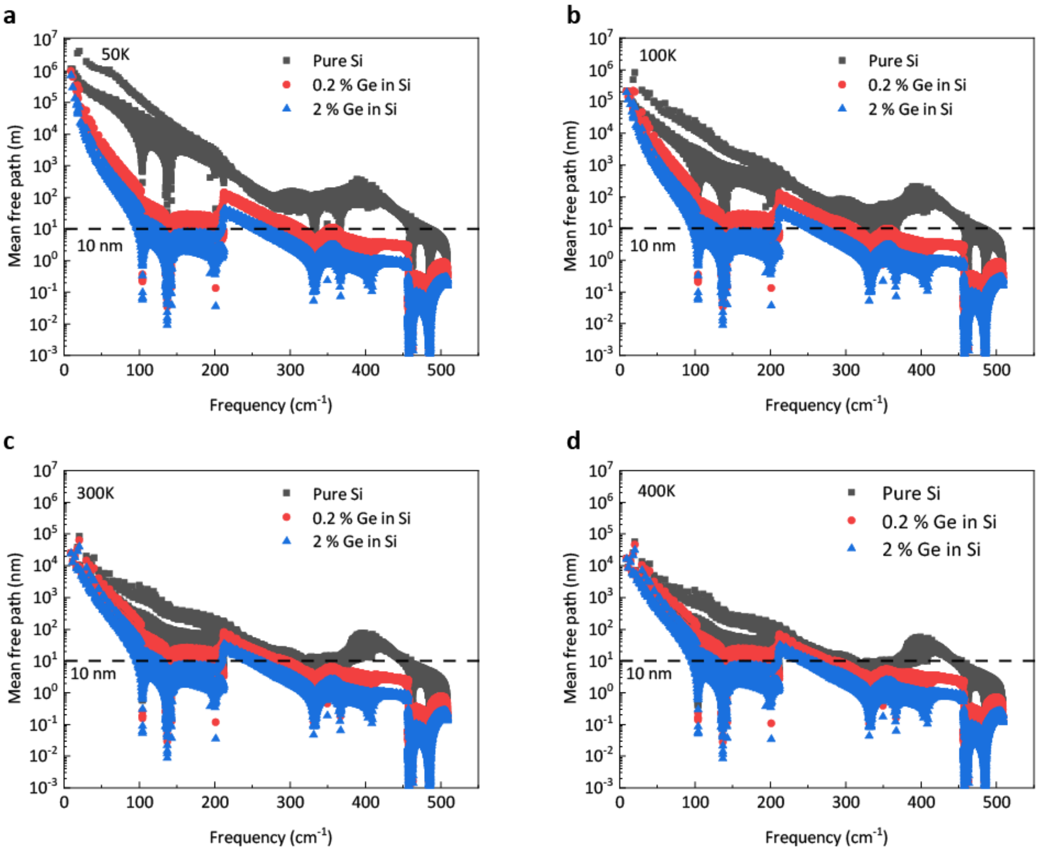

Fig. S7 | Phonon mean free path distribution of Si with Ge impurities calculated from first-principles calculations at **a**, 50 K, **b**, 100 K, **c**, 300 K, and **d**, 400 K.

Note S5. Thermal conductance enhancement of wurtzite GaN

Wurtzite GaN is widely used in power electronics [4]. We investigate thermal conductance enhancement in GaN systems with defects. We first extract phonon properties from first-principles calculations with the quantum phonon population (Bose-Einstein distribution). A supercell of 4×4×4 and the fifth nearest atom neighbor is considered to obtain the third-order anharmonic interatomic force constants. The thermal conductivity of GaN is calculated based on the single-mode relaxation time approximation method. We use 40×40×40 q-points for all temperatures to sample the Brillouin zone. The bulk thermal conductivity of GaN for different temperatures is shown in Fig. S8a. Our results agree well with those in the literature [5]. To estimate the scattering from defects, we adopt the Tamura model [6]. We adopt the mode-level phonon BTE calculations to investigate the thermal conductance enhancement by doping ^{24}Mg atoms to replace Ga atoms (system in Fig. 1(a)) with different temperatures as shown in Fig. S8b. In the main text, we introduced doping with ^{71}Ga isotopes and observed a continuous and monotonic increase in thermal conductance. This behavior can be attributed to the small mass difference between ^{71}Ga and ^{69}Ga isotopes. In contrast, a significant mass difference exists between ^{24}Mg and Ga atoms, leading to more pronounced defect scattering induced by ^{24}Mg compared to ^{71}Ga isotopes. Consequently, the thermal conductance demonstrates an initial increase followed by a subsequent decrease, as illustrated in Figure S8b.

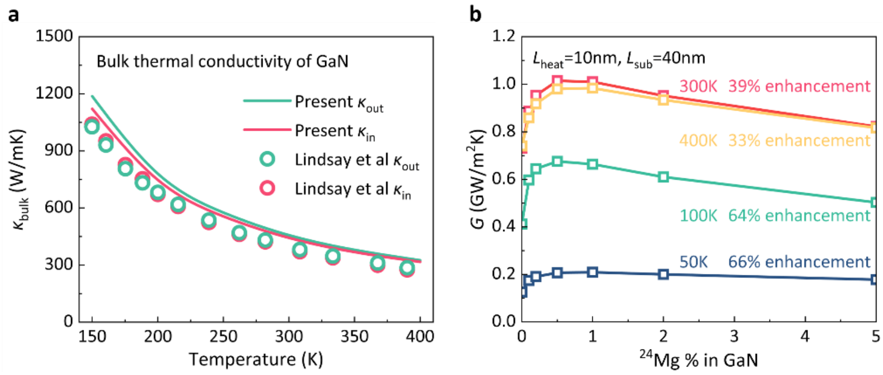

Fig. S8 | Results of wurtzite GaN systems. **a** Bulk thermal conductivity of GaN. The symbols correspond to values from the reference [5]. **b** Thermal conductance at different temperatures when doping ^{24}Mg in GaN.

Note S6. Thermal conductance enhancement of 4H-SiC

4H-SiC is also widely used in power electronics [4]. We investigate the thermal conductance enhancement in SiC systems with C^{14} isotopes. We first extract phonon properties from first-principles calculations with the quantum phonon population (Bose-Einstein distribution). A supercell of 4×4×2 and the fourth nearest atom neighbor is considered to obtain the third-order anharmonic interatomic force constants. The thermal conductivity of SiC is calculated based on the single-mode relaxation time approximation method. We use 23×23×7 q-points for all temperatures to sample the Brillouin zone, which is consistent with the reference [7]. The bulk thermal conductivity of SiC for different temperatures is shown in Fig. S9a. Our results agree well with those in the literature [7]. To estimate the scattering from defects, we adopt the Tamura model [6]. We adopt the mode-level phonon BTE calculations to investigate the thermal conductance enhancement by doping ^{10}B atoms to replace Si atoms (system in Fig. 1(a)) with different temperatures as shown in Fig. S9b. In the main text, we introduced doping with ^{14}C isotopes and observed a continuous and monotonic increase in thermal conductance. This behavior can be attributed to the small mass difference between ^{14}C and ^{12}C isotopes. In contrast, a significant mass difference exists between ^{10}B and Si atoms, leading to more pronounced defect scattering. Consequently, the thermal conductance demonstrates an initial increase followed by a subsequent decrease, as illustrated in Figure S9b.

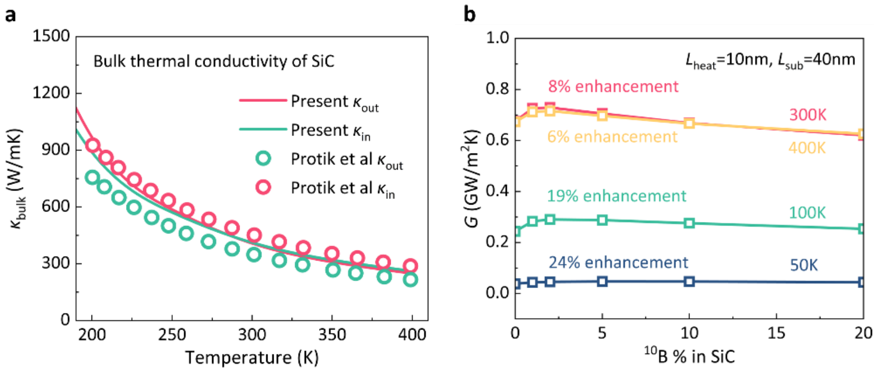

Fig. S9 | Results for 4H-SiC systems. **a** Bulk thermal conductivity of SiC. The symbols correspond to values from the reference [7]. **b** Thermal conductance at different temperatures when doping ^{10}B in SiC.

Note S7. Over-population of optical phonons

Recent studies reveal that in transistors or Raman measurements, the heat generation due to electron-phonon interactions is in spectral nonequilibrium, i.e., optical phonons (phonons with high frequency) tend to receive much more energy than acoustic phonons (phonons with low frequency) [8-11]. To study how this spectral nonequilibrium affects thermal conductance enhancement by defect scattering, we perform mode-level phonon BTE calculations with first-principles phonon properties for Si systems with a 10 nm heating zone and a 40 nm substrate. We have also conducted rigorous electron-phonon coupling calculations using the electron-phonon Wannier (EPW) package [12]. The electron-phonon coupling matrix elements are firstly calculated on the coarse meshes and are then interpolated to 100×100×100 k-point and 60×60×60 q-point meshes to calculate the electron-phonon energy generation rate with our modified codes. The calculated mode-level heat generation is shown in Fig. S10a. There are several peaks of the heat generation, which means that electrons tend to transfer energy to specific phonon modes, especially for some optical phonon modes. Meanwhile, some acoustic phonon modes also have received energy from electrons.

To quantify phonon spectral nonequilibrium, the spectral phonon temperature is usually adopted [9-11,13]. The definition of the spectral phonon temperature is $\Delta T_{\omega,p} = \int_{4\pi} e_{\omega,p,s} d\Omega / C_{\omega,p}$. When the phonons with different frequencies are in equilibrium, they have the same spectral phonon temperature. In the ballistic regime, since optical phonons receive all the energy and have poor thermal transport efficiency [9,14], optical phonons have very high temperatures that are much higher than acoustic phonons (as shown in Fig. S10b). When defect scattering is induced, the spectral nonequilibrium among phonons is largely reduced (as shown in Fig. S10b). Since acoustic phonons have higher thermal transport efficiency [9,14], the temperature is lower when the spectral nonequilibrium is smaller with a fixed heat flux.

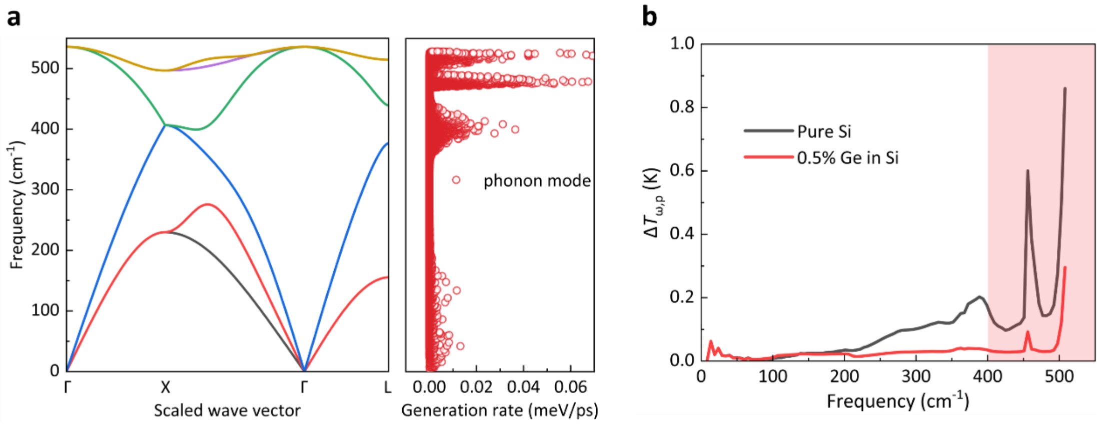

Fig. S10 | Mode-level phonon properties and spectral phonon temperature distribution. **a** Phonon dispersion and the mode-level heat generation of Si from rigorous electron-phonon coupling calculation. **b** Spectral phonon temperature distribution at the interface between the heating zone and the substrate at 100 K (Si systems with 10 nm heating zone and 40 nm substrate).

Reference

[1] Kittel, C. *Introduction to Solid State Physics* (John Wiley and Sons, New York, 2018).
[2] Giannozzi, P. *et al.* QUANTUM ESPRESSO: a modular and open-source software project for quantum simulations of materials. *J. Phys.: Condens. Matter* **21**, 395502 (2009).
[3] Broido, D. A., Malorny, M., Birner, G., Mingo, N., & Stewart, D. A. Intrinsic lattice thermal conductivity of semiconductors from first principles. *Appl. Phys. Lett.* **91**, 231922 (2007).
[4] Warzoha, R. J. *et al.* Applications and impacts of nanoscale thermal transport in electronics packaging. *J. Electron. Packag.* **143**, 020804 (2021).
[5] Lindsay, L., Broido, D. A., & Reinecke, T. L. Thermal conductivity and large isotope effect in GaN from first principles. *Phys. Rev. Lett.* **109**, 095901 (2012).
[6] Tamura, S. I. Isotope scattering of dispersive phonons in Ge. *Phys. Rev. B* **27**, 858 (1983).
[7] Protik, N. H. *et al.* Phonon thermal transport in 2H, 4H and 6H silicon carbide from first principles. *Mater. Today Phys.* **1**, 31-38 (2017).
[8] Pop, E. Energy dissipation and transport in nanoscale devices. *Nano Res.* **3**, 147-169 (2010).
[9] Vallabhaneni, A. K., Singh, D., Bao, H., Murthy, J., & Ruan, X. Reliability of Raman measurements of thermal conductivity of single-layer graphene due to selective electron-phonon coupling: A first-principles study. *Phys. Rev. B* **93**, 125432 (2016).
[10] Sullivan, S. *et al.* Optical generation and detection of local nonequilibrium phonons in suspended graphene. *Nano Lett.* **17**, 2049-2056 (2017).
[11] Zobeiri, H., Hunter, N., Wang, R., Wang, T., & Wang, X. Direct characterization of thermal nonequilibrium between optical and acoustic phonons in graphene paper under photon excitation. *Adv. Sci.* **8**, 2004712 (2021).
[12] Poncé, S., Margine, E. R., Verdi, C., & Giustino, F. EPW: Electron–phonon coupling, transport and superconducting properties using maximally localized Wannier functions. *Comput. Phys. Commun.* **209**, 116-133 (2016).
[13] Feng, T. *et al.* Spectral analysis of nonequilibrium molecular dynamics: Spectral phonon temperature and local nonequilibrium in thin films and across interfaces. *Phys. Rev. B* **95**, 195202 (2017).
[14] Chiloyan, V., Huberman, S., Maznev, A. A., Nelson, K. A., & Chen, G. Thermal transport exceeding bulk heat conduction due to nonthermal micro/nanoscale phonon populations. *Appl. Phys. Lett.* **116**, 163102 (2020).
